# GTL1 and DF1 regulate root hair growth through transcriptional repression of *ROOT HAIR DEFECTIVE 6-LIKE 4* in *Arabidopsis*

**DOI:** 10.1242/dev.159707

**Published:** 2018-02-01

**Authors:** Michitaro Shibata, Christian Breuer, Ayako Kawamura, Natalie M. Clark, Bart Rymen, Luke Braidwood, Kengo Morohashi, Wolfgang Busch, Philip N. Benfey, Rosangela Sozzani, Keiko Sugimoto

**Affiliations:** 1 RIKEN Center for Sustainable Resource Science, Yokohama 230-0045, Japan; 2 Department of Plant and Microbial Biology, North Carolina State University, Raleigh, NC 27708, USA; 3 Biomathematics Graduate Program, North Carolina State University, Raleigh, NC 27695, USA; 4 Department of Applied Biological Science, Faculty of Science and Technology, Tokyo University of Science, Noda 278-8510, Japan; 5 Gregor Mendel Institute (GMI), Austrian Academy of Sciences, Vienna Biocenter (VBC), Dr. Bohr-Gasse 3, 1030 Vienna, Austria; 6 Department of Biology, Howard Hughes Medical Institute, Duke University, Durham, NC 27695, USA

**Keywords:** Root hair, Cell growth, Trihelix transcription factor, Gene regulatory network

## Abstract

How plants determine the final size of growing cells is an important, yet unresolved, issue. Root hairs provide an excellent model system with which to study this as their final cell size is remarkably constant under constant environmental conditions. Previous studies have demonstrated that a basic helix-loop helix transcription factor ROOT HAIR DEFECTIVE 6-LIKE 4 (RSL4) promotes root hair growth, but how hair growth is terminated is not known. In this study, we demonstrate that a trihelix transcription factor GT-2-LIKE1 (GTL1) and its homolog DF1 repress root hair growth in *Arabidopsis*. Our transcriptional data, combined with genome-wide chromatin-binding data, show that GTL1 and DF1 directly bind the *RSL4* promoter and regulate its expression to repress root hair growth. Our data further show that GTL1 and RSL4 regulate each other, as well as a set of common downstream genes, many of which have previously been implicated in root hair growth. This study therefore uncovers a core regulatory module that fine-tunes the extent of root hair growth by the orchestrated actions of opposing transcription factors.

## Abstract (precis)

**Summary:** *Arabidopsis gtl1 df1* double mutants and tissue-specific overexpression of *GTL1* and *DF1* demonstrate that both GTL1 and DF1 negatively regulate root hair growth by directly repressing *RSL4*.

## INTRODUCTION

Plant cells often undergo extensive post-mitotic cell expansion and can reach up to several hundred-fold their original size (Sugimoto-Shirasu and Roberts, 2003). Controlling the final size of post-mitotically growing cells is of fundamental importance, as failure in this control can result in severe defects in plant organ growth and development (Braidwood et al., 2014; Breuer et al., 2010). Growing to an optimal size is also physiologically relevant for some specialized cell types such as root hairs, which must increase their surface area to permit better uptake of nutrients and water from the surrounding environment (Grierson et al., 2014). Root hairs grow in a polarized fashion through a localized deposition of cell wall materials at the root hair apex. This is enabled by highly directional membrane trafficking towards the growing tip of cells and subsequent exocytosis of vesicles that contain cell wall polysaccharides and cell wall proteins that need to be incorporated into newly developing cell walls (Grierson et al., 2014). As for many other cell types in plants, root hair growth is often accompanied by an increase in nuclear DNA content, or ploidy, through successive rounds of endoreduplication (Breuer et al., 2007; Sugimoto-Shirasu et al., 2005, 2002). It is also known, however, that root hair growth is ploidy independent to some degree as root hairs can elongate without changing their ploidy levels (Yi et al., 2010).

Root hairs in *Arabidopsis thaliana* (*Arabidopsis*) have served as an excellent model system for studying cell size control in plants, and molecular genetic studies over the past few decades have uncovered key regulatory mechanisms that control root hair growth. Given that root hairs are formed in a specific pattern of cell files in *Arabidopsis* roots, initiation of root hair outgrowth is regulated by a genetic program that determines cell fate (Grierson et al., 2014; Salazar-Henao et al., 2016). Key regulators that translate these developmental cues into hair initiation are the basic helix-loop-helix (bHLH) transcription factor ROOT HAIR DEFECTIVE 6 (RHD6) and its close homolog RHD6-LIKE 1 (RSL1) (Masucci and Schiefelbein, 1994; Menand et al., 2007). RHD6, together with RSL1, induces the expression of another RHD6 homolog ROOT HAIR DEFECTIVE 6-LIKE 4 (RSL4), leading to the accumulation of RSL4 proteins prior to the initiation of hair outgrowth (Datta et al., 2015; Yi et al., 2010). Remarkably, constitutive overexpression of RSL4 by the Cauliflower Mosaic Virus 35S promoter is able to maintain root hair elongation until the hair cells die (Yi et al., 2010), indicating that RSL4 is sufficient to promote root hair growth. Several recent studies have identified 132 genes that are regulated by RSL4 and have shown that RSL4 promotes the expression of these target genes by binding the root hair specific *cis*-element (RHE) in their promoter sequences (Hwang et al., 2017; Kim et al., 2006; Vijayakumar et al., 2016; Won et al., 2009; Yi et al., 2010). As expected, RSL4 target genes include those involved in cell wall biosynthesis and remodeling, vesicle trafficking, cellular signaling and metabolism, thus highlighting how the RSL4-mediated transcriptional program orchestrates various subcellular processes required for root hair growth.

Root hair growth is also fine-tuned by various hormonal and environmental cues, and several studies have shown that some of this regulation involves transcriptional upregulation of *RSL4* and subsequent activation of its downstream pathway (Franciosini et al., 2017; Marzol et al., 2017; Yi et al., 2010). Among various plant hormones, auxin is well known to enhance root hair growth (Knox et al., 2003; Lee and Cho, 2013; Pitts et al., 1998). A recent study demonstrated that several AUXIN RESPONSE FACTORs (ARFs), which are central transcriptional regulators of auxin signaling, bind the *RSL4* promoter and directly activate its expression (Mangano et al., 2017), providing the first molecular link between auxin signaling and transcriptional control of root hair development (Zhang et al., 2016). Exogenous application of ethylene also promotes root hair growth (Pitts et al., 1998) and this physiological response is accompanied by increased *RSL4* expression. Limited phosphate availability is another trigger for extended root hair growth, and this regulation also involves upregulation of *RSL4* expression (Datta et al., 2015; Yi et al., 2010).

Accumulating genetic evidence suggests that plants are also equipped with a regulatory system to actively repress root hair growth. For example, double mutants in the bHLH transcription factors *Lotus japonicas* ROOTHAIRLESS LIKE 4 (LRL4) and LRL5 produce longer root hairs compared with wild-type plants (Breuninger et al., 2016), demonstrating that root hair growth is negatively regulated by LRL4- and LRL5-dependent mechanisms. It has also been reported that ROOT HAIR SPECIFIC 1 (RHS1) and RHS10, which encode a calcium-binding protein and a receptor-like kinase, respectively, repress root hair growth, as mutating either gene results in extended hair growth (Hwang et al., 2016; Won et al., 2009). These observations thus suggest that there are multiple levels of regulation by which root hair growth can be blocked, although the exact molecular details of this control remain unknown.

We have previously reported a transcriptional mechanism that terminates cell growth in *Arabidopsis* leaf trichomes, another cell type that undergoes extensive post-mitotic cell expansion (Breuer et al., 2009). Loss-of-function mutants in the trihelix transcription factor GT-2-LIKE 1 (GTL1) develop larger trichomes than wild type, and this phenotype is associated with an increase in nuclear DNA content (Breuer et al., 2009). We showed that GTL1 terminates cell growth in a ploidy-dependent manner by repressing the expression of *CELL CYCLE SWITCH PROTEIN 52 A1* (*CCS52A1*), a key driver of plant endoreduplication (Breuer et al., 2012). Whether GTL1 acts as a general repressor of cell growth remains an unresolved issue as *gtl1* single mutants do not display obvious growth defects beyond trichomes (Breuer et al., 2009). We reasoned that there may be other transcription factors acting redundantly with GTL1. In support of this notion, another trihelix protein, called DF1, has twin trihelix binding domains that show 70% amino acid sequence identity with GTL1 (Breuer et al., 2009). In this study, we have characterized *gtl1 df1* double mutants and tissue-specific overexpression lines of *GTL1* and *DF1* to demonstrate that both GTL1 and DF1 negatively regulate root hair growth. Our data from transcriptional and chromatin immunoprecipitation (ChIP) studies suggest that GTL1 and DF1 directly repress *RSL4* as well as a set of genes previously implicated in root hair growth. We further used gene regulatory network (GRN) inference and mathematical modeling to show that GTL1 and RSL4 likely form a negative-feedback loop to cooperatively control root hair growth.

## RESULTS

### GTL1 and DF1 repress root hair growth through a ploidy-independent mechanism

**Fig. 1.**
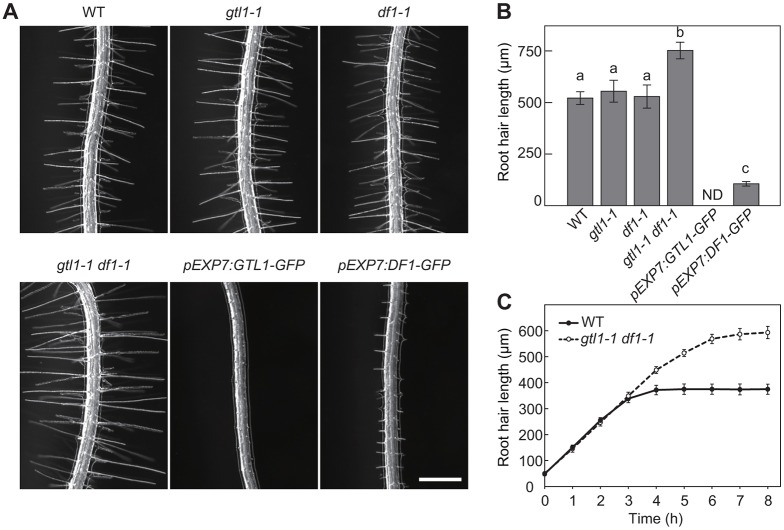
**GTL1 and DF1 repress root hair growth in *Arabidopsis*.** (A) Root hair phenotypes of wild-type, *gtl1-1*, *df1-1*, *gtl1-1 df1-1*, *pEXP7:GTL1-GFP* and *pEXP7:DF1-GFP* seedlings. Scale bar: 500 μm. (B) Quantitative analysis of root hair length. Data are mean± s.e.m. (*n*=120). Different letters indicate means that differ significantly (Tukey-Kramer test, *P*<0.05). ND indicates not detectable. (C) Time-lapse analysis of root hair growth. The rate of root hair growth is comparable between wild type and *gtl1-1 df1-1*.

To explore the functional redundancy between GTL1 and DF1, we isolated an *Arabidopsis* T-DNA insertion mutant for DF1 that resulted in a null allele (Fig. S1A). As shown in Fig. 1A, root hair growth of 7-day-old *gtl1-1* and *df1-1* single mutants is indistinguishable from wild type. In contrast, we found that root hairs in *gtl1-1 df1-1* double mutants are significantly longer compared with wild type, and their final hair length is, on average, more than 200 μm longer than wild type (Fig. 1A,B). Long root hairs in *gtl1-1 df1-1* could be caused by either faster growth and/or an extended period of their growth. Our time-lapse analysis showed that the rate of root hair growth is comparable between wild type and *gtl1-1 df1-1* but *gtl1-1 df1-1* root hairs continue to grow after wild-type root hairs halt their growth (Fig. 1C). To confirm that this growth phenotype is caused by the lack of GTL1 and DF1, we transformed *gtl1-1 df1-1* plants with *pGTL1:GTL1-GFP* or *pDF1:DF1-GFP* constructs. Introduction of either of these constructs fully rescues the hair growth phenotype, indicating that both GTL1 and DF1 contribute to the termination of root hair growth (Fig. S1B,C).

Confocal microscopy revealed that both GTL1-GFP and DF1-GFP proteins are broadly expressed along the longitudinal axis of roots, and we detected clear GFP expression in expanding root hairs (Fig. S2). To test whether overexpression of GTL1 and DF1 is sufficient to inhibit root hair growth, we ectopically expressed *GTL1-GFP* and *DF1-GFP* under the root-hair specific *EXPANSIN7* (*EXP7*) promoter (Cho and Cosgrove, 2002). The resulting *pEXP7:GTL1-GFP* and *pEXP7:DF1-GFP* plants showed an increase in the expression of *GTL1-GFP* and *DF1-GFP* by 15-fold and 30-fold, respectively (Fig. S1D). Importantly, these plants had significantly short, often undetectable, root hairs (Fig. 1A,B), demonstrating that ectopically expressed GTL1 and DF1 can repress root hair growth.

Next, we tested whether the root hair phenotypes in *gtl1-1 df1-1* mutants are accompanied by changes in ploidy levels by examining 4′6-diamidino-2-phenylindole (DAPI)-stained nuclei in fully grown root hairs. We observed that the size of DAPI-stained nuclei is comparable between wild-type and *gtl1-1 df1-1* root hairs, and quantitative analysis of DAPI-stained nuclei confirmed that the nuclear DNA content is not different between wild-type and *gtl1-1 df1-1* root hairs (Fig. S3). Consistently, unlike in trichomes, the *ccs52a1* mutation does not rescue the hair growth phenotype of *gtl1-1 df1-1* (Fig. S3C,D), indicating that CCS52A1 does not act downstream of GTL1 and DF1 in root hair development. These observations suggest that GTL1 and DF1 control root hair growth ploidy independently.

### GTL1 and DF1 directly repress *RSL4* expression in roots

**Fig. 2.**
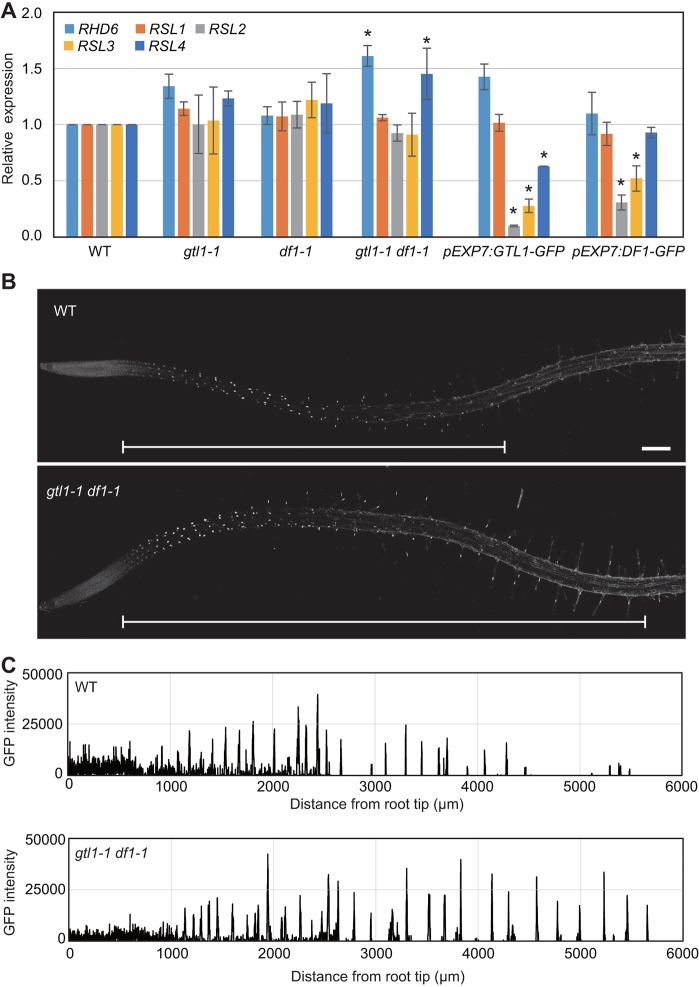
**GTL1 and DF1 repress *RSL4* expression in roots.** (A) RT-qPCR analysis of *RHD6* and *RSL1-4* in wild-type, *gtl1-1*, *df1-1*, *gtl1-1 df1-1*, *pEXP7:GTL1-GFP* and *pEXP7:DF1-GFP* roots. Expression levels are normalized against those of *UBQ10*. Data are mean±s.d. (*n*=3, biological replicates). Asterisks indicate a significant difference compared with wild type (Student's *t*-test, *P*<0.05). (B) Confocal images of RSL4-GFP in wild type and *gtl1-1 df1-1* carrying *pRSL4:RSL4-GFP*. Images are produced by maximum intensity projection from 21 *z*-stack images. White brackets indicate zones of RSL4-GFP expression. Scale bar: 250 μm. (C) The GFP signal intensity plots from B. The intensity of nuclear RSL4-GFP signals is quantified in single trichoblast cell files and plotted against distance from root tips. *gtl1-1 df1-1* roots have more GFP-positive cells compared with wild-type roots.

It has been previously shown that RSL4 promotes root hair growth in a ploidy-independent manner and without affecting the elongation rate (Yi et al., 2010). As the extended root hair growth in the *gtl1 df1* loss-of-function line resembles that of the *35S:RSL4* line, we asked whether the level of *RSL4* expression is increased in *gtl1-1 df1-1* mutants. As shown in Fig. 2A, our RT-qPCR analysis revealed that *RSL4* expression is significantly upregulated in *gtl1-1 df1-1* double mutants, suggesting that GTL1 and DF1 are required to repress *RSL4* expression. We also found that *RSL4* expression is downregulated in *pEXP7:GTL1-GFP* roots (Fig. 2A), indicating that ectopic overexpression of GTL1 is sufficient to block *RSL4* expression. Intriguingly, overexpression of DF1 does not cause significant downregulation of *RSL4* in *pEXP7:DF1-GFP* roots (Fig. 2A), implying that GTL1 has a stronger impact on *RSL4* expression. In addition, our RT-qPCR analysis revealed that, among the RHD6/RSL homologs, RHD6 expression is elevated in the *gtl1-1 df1-1* double mutants but its expression is not significantly changed in *pEXP7:GTL1-GFP* and *pEXP7:DF1-GFP* roots (Fig. 2A). Conversely, we did not detect significant changes in *RSL1*, *RSL2* and *RSL3* expression in the *gtl1-1 df1-1* double mutant, but we did observe that *RSL2* and *RSL3* are downregulated in *pEXP7:GTL1-GFP* and *pEXP7:DF1-GFP* roots (Fig. 2A). These results suggest that the primary target of GTL1 and DF1 in root hair development is *RSL4*, because among the RHD6/RSL homologs, *RSL4* shows a significant transcriptional response in both GTL1/DF1 gain-of-function and loss-of-function lines.

**Fig. 3.**
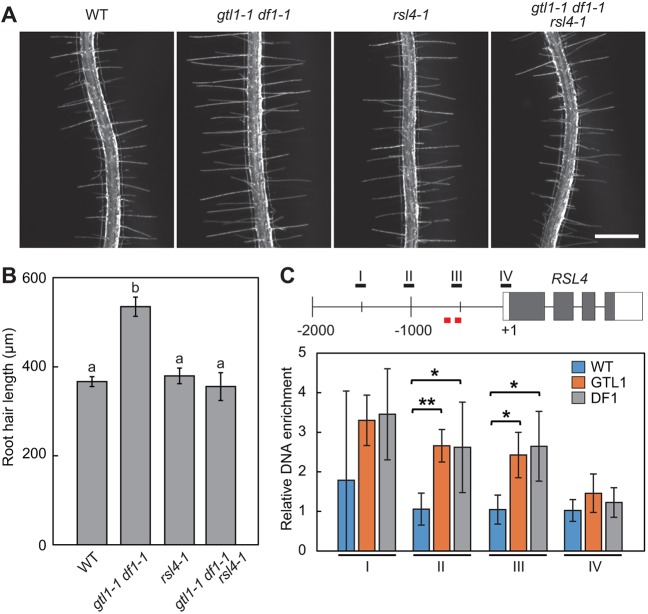
**GTL1 and DF1 directly bind the *RSL4* promoter and regulate its expression.** (A) Root hair phenotypes of wild-type, *gtl1-1 df1-1*, *rsl4-1* and *gtl1-1 df1-1 rsl4-1* seedlings. Scale bar: 500 μm. (B) Quantitative analysis of root hair length. Data are mean±s.e.m. (*n*=120). Different letters indicate means that differ significantly (Tukey-Kramer test, *P*<0.05). (C) Chromatin immunoprecipitation (ChIP) of GTL1-GFP and DF1-GFP proteins with *RSL4*. Quantitative PCR (qPCR) analysis shows the association of GTL1-GFP and DF1-GFP with the promoter of *RSL4*. ChIP experiments were performed using roots isolated from wild-type, *pGTL1:GTL1-GFP* and *pDF1-DF1-GFP* plants. Gray and white boxes, respectively, represent translated and untranslated regions of the *RSL4* transcript. Black bars highlight the positions of primer sets used for ChIP-qPCR analysis (I-IV). Red bars indicate the GT3 boxes, 5′-GGTAAA-3′ at −556 bp and 5′-TTTACC-3′ at −785 bp, previously described as binding motifs for GTL1 (Breuer et al., 2012). Average enrichment of qPCR products was normalized against corresponding input DNA. Data are mean±s.d. (*n*=3). Asterisks indicate a significant difference compared with wild type (Student's *t*-test, **P<0.01, *P<0.05).

To investigate whether transcriptional activation of *RSL4* leads to the increased levels of RSL4 proteins in root hairs, we introduced the *pRSL4:GFP-RSL4* construct (Yi et al., 2010) into *gtl1-1 df1-1* double mutants and examined the GFP-RSL4 accumulation by confocal microscopy. As previously reported, GFP-RSL4 proteins accumulate in trichoblast cells prior to root hair initiation and their accumulation sharply declines when hair cells complete their outgrowth in the wild-type background (Yi et al., 2010 and Fig. 2B). In sharp contrast, we found that GFP-RSL4 detection persists much longer in maturing root hairs in the *gtl1-1 df1-1* background. Accordingly, our quantitative analysis showed that the number of RSL4-GFP-expressing cells in trichoblast cell files is significantly increased in *gtl1-1 df1-1* roots (Fig. 2B,C). In order to test whether RSL4 upregulation is responsible for the extended hair growth in *gtl1-1 df1-1*, we introduced the *rsl4-1* mutation into the *gtl1-1 df1-1* mutant background. As shown in Fig. 3A,B, introduction of *rsl4-1* into *gtl1-1 df1-1* rescues the root hair phenotype in *gtl1-1 df1-1*, further substantiating that RSL4 is a key regulator of root hair growth acting downstream of GTL1 and DF1. We subsequently investigated whether GTL1 and DF1 directly bind the *RSL4* promoter by immunoprecipitating GTL1-GFP and DF1-GFP proteins from 7-day-old *pGTL1:GTL1-GFP* and *pDF1:DF1-GFP* roots. Our chromatin immunoprecipitation (ChIP) followed by quantitative PCR (ChIP-qPCR) analysis showed an enrichment of both GTL1-GFP and DF1-GFP around 500-1000 bp upstream of the *RSL4* start codon containing two binding motifs for GTL1 (Breuer et al., 2012) (Fig. 3C). To further support these results, we co-bombarded the *p35S:GTL1* construct and *pRSL4:LUC* promoter into *Arabidopsis* MM2D culture cells and tested whether GTL1 can repress the *RSL4* promoter activity. As shown in Fig. S4, application of *p35S:GTL1* significantly suppresses the expression of *pRSL4:LUC* compared with the vector control. These results thus suggest that GTL1 and DF1 directly bind the promoter region of *RSL4* to repress its expression.

### GTL1 and DF1 act in a parallel pathway with auxin signaling to regulate root hair development

**Fig. 4.**
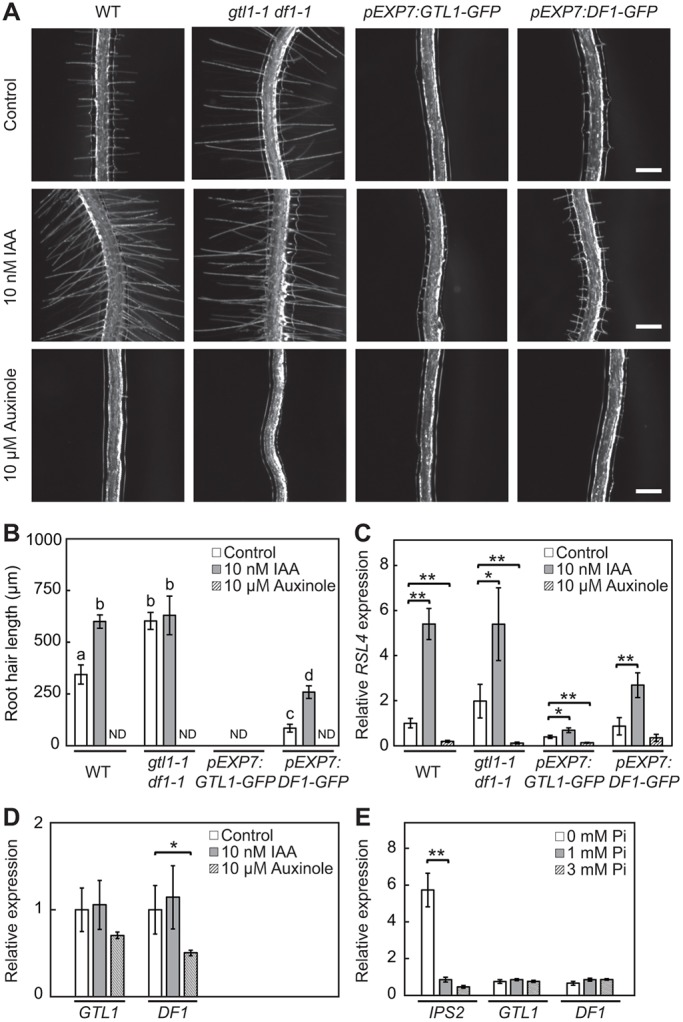
**GTL1 and DF1 regulate root hair growth in a parallel pathway to auxin signaling.** (A) Root hair phenotypes of wild-type, *gtl1-1 df1-1*, *pEXP7:GTL1-GFP* and *pEXP7:DF1-GFP* seedlings treated with 10 nM IAA or 10 μM auxinole. Scale bars: 300 μm. (B) Quantitative analysis of root hair length. Data are mean±s.e.m. (*n*=60). Different letters indicate means that differ significantly (Tukey-Kramer test, *P*<0.05). ND indicates not detectable. (C) RT-qPCR analysis of *RSL4* in wild-type, *gtl1-1 df1-1*, *pEXP7:GTL1-GFP* and *pEXP7:DF1-GFP* roots treated with 10 nM IAA or 10 μM auxinole. (D) RT-qPCR analysis of *GTL1* and *DF1* in wild-type roots treated with 10 nM IAA or 10 μM auxinole. Expression levels are normalized against those of *UBQ10* in C,D. Asterisks indicate a significant difference compared with a control treated with DMSO and ethanol (Student's *t*-test, **P<0.01, *P<0.05). (E) RT-qPCR analysis of *GTL1* and *DF1* in wild type grown under 0 mM, 1 mM and 3 mM phosphate (Pi) condition. *INDUCED BY PI STARVATION 2* (*IPS2*) was used as the positive control for phosphate response. Asterisks indicate a significant difference compared with the control condition (1 mM) (Student's *t*-test, **P<0.01).

Auxin is known to promote root hair growth by activating the expression of *RSL4* (Mangano et al., 2017; Yi et al., 2010). To investigate whether GTL1 and DF1 are also involved in auxin-induced root hair growth, we first studied the auxin response in *gtl1-1 df1-1* mutants, as well as in *GTL1* and *DF1* overexpression lines. As previously reported (Grierson et al., 2014), wild-type plants grown in the presence of 10 nM indole-3-acetic acid (IAA) display significantly longer root hairs compared with control plants (Fig. 4A,B). In contrast, root hair growth is comparable between control and IAA-treated *gtl1-1 df1-1* mutants (Fig. 4A,B). We next tested whether IAA can rescue the compromised hair growth in *pEXP7:GTL1-GFP* and *pEXP7:DF1-GFP* plants but found that IAA has little to no impact on root hair growth in these overexpression lines (Fig. 4A,B). Conversely, the application of an auxin inhibitor, auxinole (Hayashi et al., 2012), completely suppresses root hair growth in wild type, *gtl1-1 df1-1* and *GTL1* or *DF1* overexpression lines (Fig. 4A,B). These results together suggest that GTL1 and DF1 likely function independently from auxin signaling in controlling root hair growth.

It has been demonstrated previously that the application of another synthetic auxin, 1-naphthaleneacetic acid (NAA), upregulates *RSL4* expression (Yi et al., 2010). Consistently, our RT-qPCR analysis revealed that IAA promotes *RSL4* expression and that auxinole strongly suppresses its expression in both wild-type and *gtl1-1 df1-1* roots (Fig. 4C). Our RT-qPCR data, in addition, showed that overexpression of *GTL1* and *DF1* counteracts this auxin-induced *RSL4* upregulation as the level of *RSL4* expression is strongly reduced in *pEXP7:GTL1-GFP* and *pEXP7:DF1-GFP* plants, regardless of treatment with IAA or auxinole (Fig. 4C). Unlike *RSL4*, the expression of *GTL1* and *DF1* does not seem to be affected by IAA, although their expression tends to be lower in plants treated with auxinole (Fig. 4D). These results, therefore, support the hypothesis that GTL1 and DF1 control root hair growth via regulation of *RSL4* in a parallel pathway with auxin. In addition to auxin, phosphate (Pi) availability is well known to affect root hair growth (Datta et al., 2015; Yi et al., 2010). Similar to auxin treatment, however, our RT-qPCR data showed that phosphate conditions do not change the expression of *GTL1* and *DF1* (Fig. 4E), suggesting that GTL1 and DF1 do not act via the phosphate-mediated control of root hair growth.

### GTL1 and DF1 directly regulate a subset of RSL4 target genes

Recent studies have identified 132 genes that might be directly activated by RSL4 in *Arabidopsis* roots (Hwang et al., 2017; Vijayakumar et al., 2016; Won et al., 2009; Yi et al., 2010). Having uncovered that GTL1 and DF1 repress *RSL4* expression, we sought to determine how much the GTL1/DF1-regulated gene regulatory network overlaps with that of RSL4. To identify genes regulated by GTL1 and DF1 in root hairs, we collected root hair cells overexpressing *GTL1-GFP* and *DF1-GFP*, respectively, from *pEXP7:GTL1-GFP* and *pEXP7:DF1-GFP* plants by fluorescence-activated cell sorting. We also collected root hair cells from *gtl1-1 df1-1* plant expressing the root hair specific *pEXP7:NLS-GFP* marker (Ikeuchi et al., 2015) and compared their transcriptional profile using the Affymetrix ATH1 microarray. We defined GTL1 or DF1 response genes as those that show more than a twofold change of gene expression in *gtl1-1 df1-1* plants compared with *pEXP7:GTL1-GFP* or *pEXP7:DF1-GFP* plants (*P*<0.05). Using this criterion, we identified 1552 genes upregulated and 1359 genes downregulated by GTL1 (Table S1). Comparing these GTL1 response genes with the 132 RSL4-activated genes, we found only five (3.8%) of the RSL4-induced genes are upregulated by GTL1 (Fig. S5A). On the other hand, 36 (27.2%) of the RSL4-induced genes are repressed by GTL1 (Fig. S5A and Table 1), strongly suggesting that GTL1 and RSL4 share many common targets but they act in opposing manners. For DF1, we identified 755 activated and 918 repressed genes, but only 7 (5.3%) of the repressed genes are RSL4 response genes (Fig. S5A, Table S1). Given that all seven of these RSL4 response genes are also repressed by GTL1 (Fig. S5B), we focused our remaining analysis on the 36 genes commonly regulated by GTL1 and RSL4.

**Table 1.**
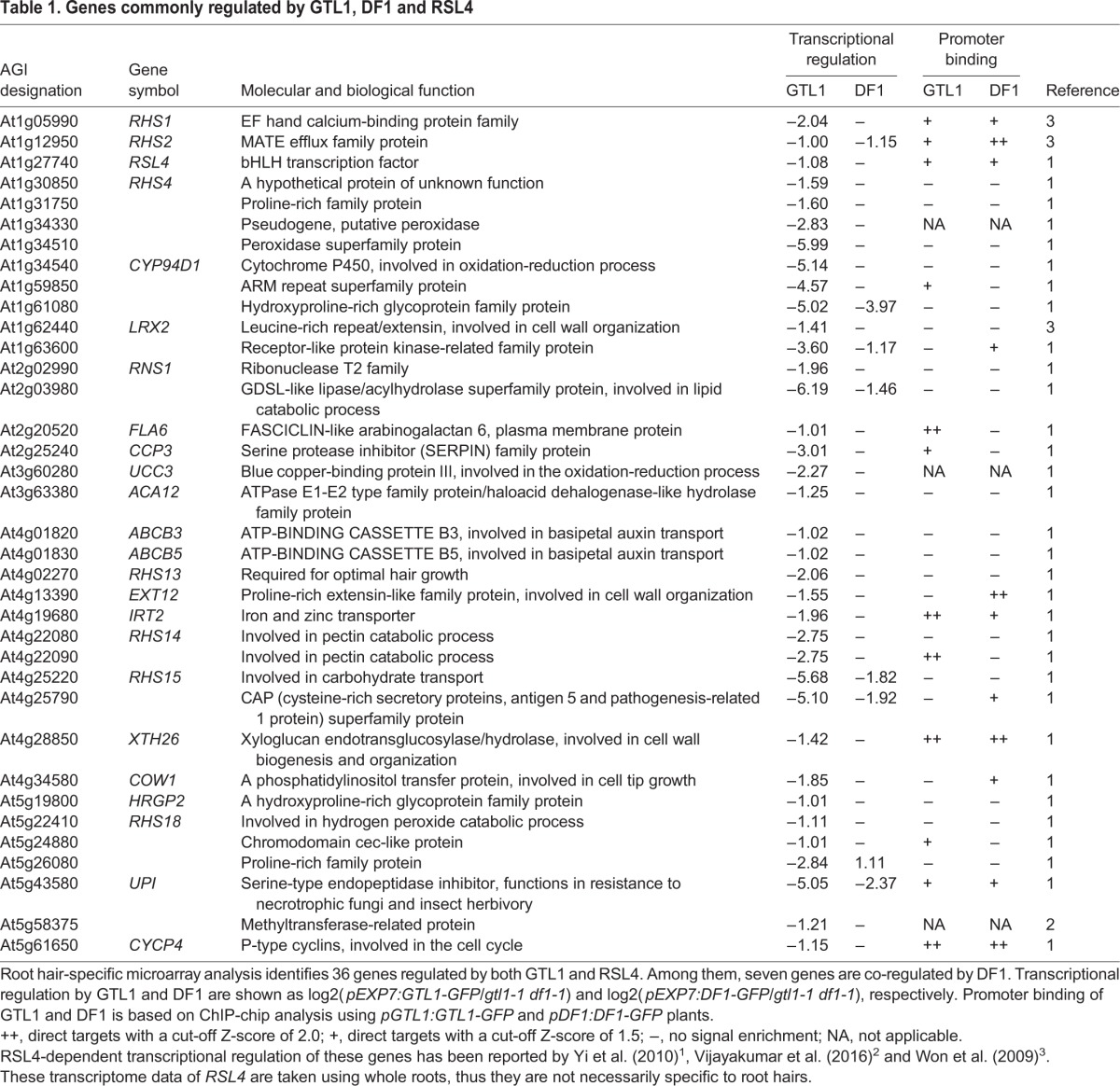
**Genes commonly regulated by GTL1, DF1 and RSL4**

The 36 GTL1-RSL4 common downstream genes include those that encode proteins associated with cell wall biosynthesis and remodeling (e.g. *EXT12*, *XTH26* and *LRX2*), those likely acting in cellular signaling (e.g. *RHS1*, RLK genes and *ACA12*), and those with functions in membrane trafficking or membrane transport (e.g. *ABCB3*, *ABCB5*, *IRT2* and *COW1*) (Table 1), suggesting that GTL1 and RSL4 regulate a wide range of subcellular processes underlying root hair growth. Having uncovered a substantial overlap between RSL4-activated genes and GTL1/DF1-repressed genes, we wanted to test whether GTL1 and DF1 also directly bind any of these genes. To achieve this, we performed a whole-genome ChIP-chip analysis using *pGTL1:GTL1-GFP* and *pDF1:DF1-GFP* roots. We identified 9200 putative direct targets for GTL1 and 7803 putative targets for DF1, 5208 (66.7% of DF1 targets) of which are common between GTL1 and DF1 (Z-score>1.5) (Fig. S6A and Table S2). Using these ChIP-chip data, we found that 12 (33.3%) of the 36 targets shared between GTL1 and RSL4 are directly bound by GTL1 (Table 1). We also found that DF1 directly binds 11 (30.6%) of the 36 genes but only four of these 11 (36.4%) are transcriptionally repressed by DF1 (Table 1), implying that the physical binding of DF1 to the target promoter sequence does not necessarily cause downstream transcriptional repression. These data demonstrate that GTL1, DF1 and RSL4 control root hair growth by regulating partially overlapping transcriptional pathways.

### GTL1 and RSL4 control root hair growth through a negative-feedback loop

**Fig. 5.**
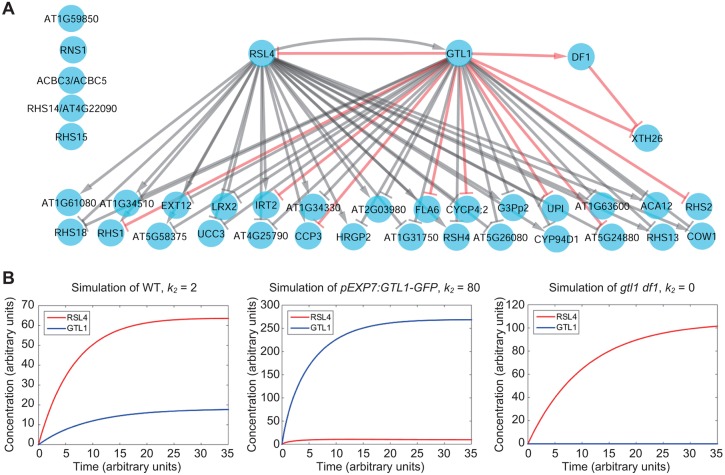
**Modeling predicts that GTL1 and RSL4 form a negative-feedback loop and regulate a set of common target genes.** (A) Gene regulatory network (GRN) of GTL1, RSL4 and their shared downstream targets. Transcriptional activation and repression are indicated by arrows and bars, respectively. A time-course transcriptome of elongation and differentiation zones was used to predict the transcriptional activation and repression of target genes. Red edges represent direct promoter binding validated using ChIP-chip data. (B) Model simulation of *GTL1* and *RSL4* dynamics in wild-type (left), *pEXP7:GTL1-GFP* (middle) and *gtl1-1 df-1* mutant (right) roots. Red and blue lines represent model solutions for the RSL4 and GTL1 equations, respectively. The value of *k_2_*, the production rate of GTL1, was varied from the wild-type case so that the steady-state values of GTL1 in *pEXP7:GTL1-GFP* and *gtl1-1 df-1* roots meet the value estimated from our RT-qPCR data.

**Fig. 6.**
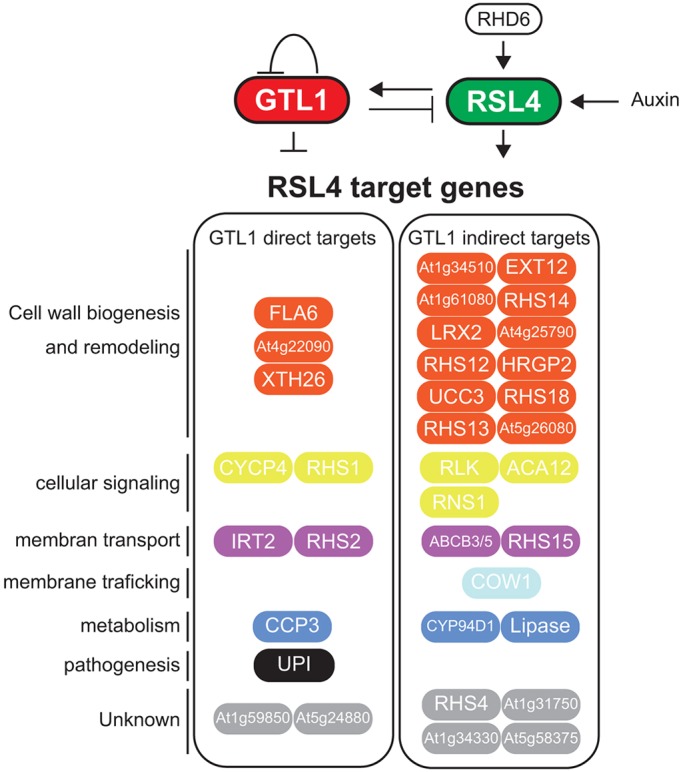
**A schematic diagram of the proposed transcriptional network regulating root hair growth in *Arabidopsis*.** RHD6 induces *RSL4* expression to promote root hair outgrowth. GTL1 binds the *RSL4* promoter and directly represses its expression to terminate root hair growth. RSL4, in addition, activates *GTL1*, thus forming a negative-feedback loop. RSL4 and GTL1 regulate, both directly and indirectly, a set of common downstream genes involved in cell wall biosynthesis and remodeling, cellular signaling, membrane transport, membrane trafficking, metabolism, pathogenesis and unknown functions. Auxin promotes root hair growth by activating *RSL4* but not *GTL1*.

Our transcriptome and ChIP-chip data suggest that GTL1 and DF1 directly regulate *RSL4* and a subset of its downstream targets. To further investigate the regulatory roles of GTL1 and DF1, we employed gene network inference with ensemble of tree 3 (GENIE3) (Huynh-Thu et al., 2010) and inferred a gene regulatory network (GRN) among GTL1, DF1, RSL4 and their 36 common target genes using our expression data from *GTL1* and *DF1* mutants (*gtl1-1*, *df1-1*, *gtl1-1 df1-1*) and from *GTL1* and *DF1* overexpression lines (*pEXP7:GTL1-GFP* and *pEXP7:DF1-GFP*). We also obtained the sign (positive/negative) of regulation using time-course RNA-seq data from 3-, 4-, 5-, 6- and 7-day-old wild-type roots (de Luis Balaguer et al., 2017). Our GRN predicts that GTL1 regulates 30 (83%) of the 36 downstream targets, 28 of which (93%) are also predicted to be regulated by RSL4 (Fig. 5A). These data further substantiate that GTL1 and RSL4 co-regulate many of these root hair genes to control root hair growth. Of the 30 targets GTL1 is predicted to regulate, our sign algorithm predicts that 29 of those (97%) are repressions (Fig. 6A). In contrast, our sign algorithm predicts that of the 28 genes regulated by RSL4, 21 (75%) are activated by RSL4 (Fig. 5A). In agreement with DF1 not playing a central role in transcriptional repression of root hair genes, we observed that DF1 has only one predicted downstream target, *XTH26*, which is not predicted to be regulated by RSL4 (Fig. 5A).

Our GRN, in addition, predicted that RSL4 and GTL1 form a negative-feedback loop (Fig. 5A). As positive regulation of *GTL1* by RSL4 has also been reported by a previous study using *RSL4*-inducible lines (Vijayakumar et al., 2016), we hypothesized that this mutual regulation is important for establishing proper root hair growth. To explore this reciprocal regulation further, we developed a mathematical model between RSL4 and GTL1, and tested whether their protein levels can reach a steady state. In addition to the negative-feedback loop between GTL1 and RSL4, our ChIP-chip data and transient promoter-LUC data suggested that GTL1 is able to repress itself (Table S2 and Fig. S4). Thus, we incorporated the auto-repression of GTL1 into the model. We incorporated several parameters, such as production and degradation rates of GTL1 and RSL4, to estimate the levels of GTL1 and RSL4 over time. Our model predicted that for certain parameter values, there exists a steady state for GTL1 and RSL4, likely representing the wild-type situation where root hairs grow to a constant size (Fig. 5B and Fig. S7A). We subsequently used a sensitivity analysis to identify the most important parameters in the model that influence the steady states of GTL1 and RSL4. Using Sobol decomposition, we found that the production rate of RSL4, *k_1_*, and the production rate of GTL1, *k_2_*, are the two most important parameters in the model (Fig. S7B and Table S4).

To test whether RSL4 and GTL1 alone are able to account for the observed root hair phenotypes, we subsequently modified the values of their production rates (*k*_1_ and *k*_2_, respectively) and examined the changes in the steady state. First, to simulate GTL1 overexpression, we increased the production rate of GTL1 (*k*_2_) until the steady state value of GTL1 was 15-fold higher than in wild type, as estimated by our RT-qPCR data on *pEXP7:GTL1-GFP* plants (Fig. S1D). Our model predicts that the steady state of RSL4 is 16% of the value in wild type (Fig. 5B, Fig. S7A), suggesting that, as GTL1 increases, RSL4 decreases and causes shorter root hairs, which is the phenotype we observed in *pEXP7:GTL1-GFP* plants (Fig. 1A). Next, to simulate the *gtl1-1 df1-1* mutant, we set the production rate of GTL1 (*k*_2_) to 0, as our RT-qPCR data show that GTL1 expression is effectively zero in *gtl1-1 df1-1* (Fig. S1D). Our model shows that in this scenario the steady state of RSL4 is 1.5-fold higher than in wild type, which is supported by our RT-qPCR data (Figs 2A and 5B, Fig. S6A). These results thus suggest how the expression levels of GTL1 and RSL4 can be altered to determine the final length of root hairs.

## DISCUSSION

### The GTL1-RSL4 module in the GRN of root hair growth

In this study we demonstrate that the final size of root hair cells is regulated by coordinated action of GTL1 and RSL4, which serve as a repressor and activator, respectively, of root hair growth. Previous studies have identified several negative regulators of root hair growth (Breuninger et al., 2016; Hwang et al., 2016), but how they suppress root hair growth is not known. Our data uncover an effective strategy to fine-tune root hair growth that relies on the negative regulation of RSL4, which is one of the central hubs in the transcriptional network of root hair growth (Datta et al., 2015; Marzol et al., 2017; Yi et al., 2010). It is interesting that GTL1 and DF1 control only the duration of root hair growth and not the rate of hair growth. These observations are consistent with previous data showing that the ectopic RSL4 expression does not change the rate of hair growth and only prolongs hair growth (Yi et al., 2010). These results thus suggest that these two parameters of root hair growth can be mechanistically uncoupled and, although the GTL1-RSL4 module controls the extent of root hair growth, other as yet unknown pathways control the rate of root hair growth. As *gtl1-1 df1-1* double mutants, but not their single mutants, display root hair phenotypes (Fig. 1A,B), we hypothesized that GTL1 and DF1 work redundantly on root hair development. In agreement with this, there is a large overlap between GTL1- and DF1-response genes (Fig. S5B and Table S1). DF1, however, has less of an impact on *RSL4* expression than GTL1 (Fig. 2A), and our GRN predicts that the contribution of DF1 to regulating known root hair genes is much less than GTL1 (Fig. 5A). These results thus suggest that DF1 may function as a backup system for GTL1.

Our data show that GTL1 and RSL4 regulate a set of common downstream genes (Figs 5 and 6), which allows robust control of target gene expression. Another important aspect of our root hair GRN is that GTL1 and RSL4 form a negative-feedback loop that results in steady-state levels of their expression, thus permitting consistent hair growth in wild-type plants (Fig. 5). In addition to *RSL4*, our RT-qPCR analysis shows that *RHD6* is also upregulated in *gtl1-1 df1-1* mutants (Fig. 2A), suggesting that RHD6 is another potential target of GTL1 and/or DF1. Indeed, our ChIP-chip data support this notion as both GTL1 and DF1 bind the promoter sequence of *RHD6* (Table S2). Given that RHD6 directly binds *RSL4* (Yi et al., 2010), this suggests a feed-forward loop between GTL1, RHD6 and RSL4. Interestingly, expression levels of *Lotus japonicus ROOTHAIRLESS1-LIKE1* (*LRL1*), which encodes a bHLH transcription factor that acts as an activator of root hair growth (Karas et al., 2009; Lin et al., 2015), is also correlated with *GTL1* and *DF1* expression based on our transcriptome data (Table S1). Thus, the GTL1-RSL4 GRN we unveiled in this study may be expanded in future studies to include RHD6, LRL1 and perhaps other root hair regulators to further understand the core transcriptional modules that control root hair growth.

### Physiological roles of GTL1 in root hair growth

We found that the majority of the 36 GTL1-RSL4 common targets are genes related to cell wall biosynthesis and remodeling (Table 1 and Fig. 6). We believe this is reasonable as cell-wall construction is directly involved in cell expansion. In addition, RHS1, one of the GTL1-RSL4 common downstream genes (Table 1 and Fig. 6), is a calmodulin-like protein that is thought to regulate root hair growth through Ca^2+^ signaling (Won et al., 2009). Another GTL1-RSL4 target, CAN OF WORMS 1 (COW1), is implicated in membrane trafficking and required for root hair tip growth, as its loss-of-function mutation causes shorter root hairs (Böhme et al., 2004; Grierson et al., 1997). GTL1-RSL4 common targets also include genes associated with several other physiological functions. IRON REGULATED TRANSPORTER 2 (IRT2), for example, regulates iron uptake from soil (Vert et al., 2009), and UNUSUAL SERINE PROTEASE INHIBITOR (UPI) is involved in pathogen resistance (Laluk and Mengiste, 2011). Since root hairs result from outgrowth of the root epidermis, they directly interact with the microenvironment of the soil. It is thus likely that these microenvironments influence the extent of root hair growth, and this interaction also needs to be regulated by GTL1. Consistently, our GO analysis of GTL1 targets suggests that GTL1 affects ‘response to stimulus’, ‘response to stress’ and ‘response to chemical stimulus’ (Fig. S5B), suggesting that GTL1 regulates diverse arrays of physiological processes associated with root hair growth.

### Conclusions

In this study we demonstrate that the final size of root hair cells is regulated by coordinated action of GTL1 and RSL4, which serve as a repressor and activator, respectively, of root hair growth. Our mathematical analysis suggests that GTL1-RSL4 functions as a core module in root hair growth and regulates cell expansion, as well as several other physiological responses such as nutrient uptake and pathogen response. Our data, in addition, suggest that other previously described root hair regulators, such as RHD6 and LRL1, may function within a larger GRN that controls root hair growth downstream of the GTL1-RSL4 module.

## MATERIALS AND METHODS

### Plant materials and growth conditions

The *gtl1-1*, *ccs52a1-2*, *rsl4-1*, *pGTL1:GTL1-GFP*, *pRSL4:RSL4-GFP*, *pEXP7:GTL1-GFP* and *pEXP7:NLS-GFP* lines have been previously described (Breuer et al., 2009, 2012; Ikeuchi et al., 2015; Yi et al., 2010). The *df1-1* (SALK_106258) mutant was obtained from the Arabidopsis Biological Resource Center. All mutants and transgenic lines used in this study were in the Columbia-0 background. Plants were grown on half-strength Johnson media with 6 g/l gelzan (Sigma) and final concentration of phosphate adjusted to 1 mM (Johnson et al., 1957; Ma et al., 2001). For auxin and auxin inhibitor treatment, a solution of 1 mM IAA and 30 mM auxinole (Hayashi et al., 2012) in mixed DMSO and ethanol were added to the Johnson media to final concentrations of 10 nM and 10 μM, respectively. Phosphate media were prepared based on recipes of Ma et al. (2001).

### Plasmid construction and plant transformation

For the construction of the *pDF1:DF1-GFP* vector, a 4900 bp genomic fragment of the *DF1* locus was amplified from the BAC clone F7O12 and cloned into the *pENTR/D-TOPO* vector (Invitrogen). A *Sma*I restriction site was introduced upstream of the translational stop codon by site-directed mutagenesis. A *Sma*I-digested GFP fragment was then inserted to create a C-terminal translational fusion construct and the resulting construct was cloned into the pGWB1 binary vector (Nakagawa et al., 2007). The *pEXP7:DF1-GFP* vector was generated by combining *pDONR-pEXP7* and *pENTR-DF1-GFP* together with the R4pGW501 destination vector (Nakagawa et al., 2008) as described by Ikeuchi et al. (2015). A set of primers used for PCR amplification is provided in Table S5. All plant transformation was carried out using the floral dip method (Clough and Bent, 1998).

### RNA extraction and RT-qPCR analysis

Total RNA was extracted from 7-day-old roots using an RNeasy Plant Mini Kit (Qiagen). Extracted RNA was reverse transcribed using a PrimeScript RT-PCR kit with DNase I (Perfect Real Time) (Takara) in accordance with the accompanying protocol. Transcript levels were determined by RT-qPCR using a THUNDERBIRD SYBR qPCR Mix kit (Toyobo) and an Mx399P QPCR system (Agilent). The expression of the *UBQ10* gene was used as a reference (Shibata et al., 2013). A set of primers used for RT-qPCR is provided in Table S5.

### Fluorescence-activated cell sorting and microarray analysis

To identify genes regulated by GTL1 and DF1 in root hairs, GFP-positive cells were sorted from 5-day-old wild-type and *gtl1-1 df1-1* roots carrying *pEXP7:NLS-GFP*, as well as from *pEXP7:GTL1-GFP* and *pEXP7:DF1-GFP* roots. The root tips were dissected at ∼0.5 cm from root tips and GFP-positive protoplasts were isolated by fluorescence-activated cell sorting, following the protocol described by Sozzani et al. (2010). Total RNA was extracted from three biological replicates and labeled probes were used for hybridization on ATH1 chips (Affymetrix). Microarray data were analyzed using R software and the gcRMA implementation with AffylmGUI (Wettenhall et al., 2006) of the Bioconductor package as previously described (Morohashi and Grotewold, 2009; Sozzani et al., 2010). Microarray data have been deposited in Gene Expression Omnibus (https://www.ncbi.nlm.nih.gov/geo/) under accession number GSE103917.

### ChIP-chip and ChIP-qPCR analysis

ChIP-chip experiment was performed using 5-day-old *pGTL1:GTL1-GFP* and *pDF1:DF1-GFP* roots as previously described (Sozzani et al., 2010). GTL1-GFP and DF1-GFP proteins were immunoprecipitated from three biological replicates using antibodies against GFP (ab290, Abcam). Bovine serum albumin was used as a negative control. Labeled DNA was hybridized to a custom-made *Arabidopsis* promoter microarray (Sozzani et al., 2010). ChIP-chip data were analyzed as previously described (Sozzani et al., 2010). Relatively low Z-scores were chosen to decrease the number of false negatives and identify meaningful overlaps with the expression data. ChIP-chip data have been deposited in Gene Expression Omnibus (https://www.ncbi.nlm.nih.gov/geo/) under accession number GSE104010. ChIP-qPCR analysis was performed using 7-day-old *pGTL1:GTL1-GFP* and *pDF1:DF1-GFP* roots as previously described (Rymen et al., 2017). Data were normalized against input DNA and shown as relative enrichment of DNA immunoprecipitated at the *TA3* retrotransposon locus (Yamaguchi et al., 2014). A set of primers used for ChIP-qPCR is provided in Table S5.

### Promoter-luciferase assay

To construct the firefly luciferase (LUC) reporter vector, a 1.8 kb promoter of *GTL1* was amplified by PCR and introduced into the LUC reporter vector (Ohta et al., 2001), as reported by Rymen et al. (2017). For the construction of the effector vector, the coding sequence of *GTL1* was amplified by PCR and cloned into the *p35SSG* vector (Mitsuda et al., 2005) using the *Sma*I site located between the CaMV p35S promoter-Omega and the NOS terminator sequence of the *p35SSG* vector. The set of primers used for PCR amplification is provided in Table S5.

The *p35S:GTL1* and the empty *p35SSG* vectors were used as an effector and control vector, respectively. The *pRSL4:LUC* and *pGTL1:LUC* vectors were used as reporters. As an internal control, the *pPTRL* vector, which drives the expression of a Renilla LUC gene under the control of the CaMV p35S promoter, was used. The constructions were introduced into *Arabidopsis* MM2D culture cells (Menges and Murray, 2002) using a gold particle bombardment system. Luciferase activities were quantified using a Mithras LB940 microplate luminometer (Berthold Technologies) according to the protocol described previously (Hiratsu et al., 2002).

### Microscopy

Root hair phenotypes were recorded using a Leica M165 FC dissection microscope equipped with a digital Leica DFC 7000T camera. The length of 120 root hairs from at least six seedlings were quantified for each genotype with ImageJ version 1.50a. For auxin and auxin inhibitor treatment, the lengths of 60 root hairs from at least six seedlings were quantified. To quantify the rate of root hair growth, hair growth of 7-day-old wild-type and *gtl1-1 df1-1* seedlings was recorded every 1 h and root hair length was quantified by ImageJ. To estimate the ploidy level of nuclei, nuclei were stained with DAPI (Partec) and visualized using an Olympus BX51 fluorescence microscope equipped with a digital Olympus DP70 camera and Olympus DP Manager software version 1.2.1.107 (Zhang and Oppenheimer, 2004). Fluorescence signals of over 40 root cap nuclei and 120 root hair nuclei from at least five seedlings were quantified per genotype with ImageJ as previously described (Ikeuchi et al., 2015). Expression patterns of GTL1-GFP, DF1-GFP and RSL4-GFP proteins were examined using a SP5 confocal laser scanning microscope (Leica). Recorded images were exported as a 16-bit TIFF image. Fluorescence intensity was quantified using ImageJ. To produce a merged image from multiple images, ‘photomerge’ was applied on Photoshop CS3 Extended (Adobe). Contrast and brightness were adjusted using Photoshop.

### Gene regulatory network inference

Gene network inference with ensemble of trees 3 (GENIE3) (Huynh-Thu et al., 2010) was used to predict the downstream targets of GTL1 and RSL4. GENIE3 uses regression tree inference to build the GRN that best fits the experimental data. Microarray data from *gtl1-1*, *df1-1, gtl1-1 df1-1*, *pEXP7:GTL1-GFP* and *pEXP7:DF1-GFP* were used to infer 10,000 directed regression trees using the Random Forest method (Breiman, 2001), and the trees were then averaged to form the final GRN. Once the final GRN was obtained, a threshold was set on the number of edges to increase the precision. The threshold was chosen using ChIP-chip data to validate the downstream targets of GTL1 in the network. The number of edges was chosen as floor (1.65×36)=59 edges (1.65×number of genes), as this number resulted in the highest precision for the network (Table S3).

A time-course RNAseq dataset of the root meristem (de Luis Balaguer et al., 2017) was used to determine the sign of the regulation from gene A to gene B. According to the first-order Markov assumption, if gene B increases (or decreases) at one time point after gene A increases (decreases), the regulation at that time point is assumed to be positive. Similarly, if gene B increases (or decreases) after gene A decreases (increases), the regulation at that time point is assumed to be negative (de Luis Balaguer et al., 2017). The Markov assumption was used to calculate the sign for all the time points, and then the majority sign was used for the edge.

### Mathematical modeling and sensitivity analysis

The mathematical model consisted of two ordinary differential equations: one measuring RSL4 concentration (*R*) and the other measuring GTL1 concentration (*G*). It was assumed that transcription and translation happen quickly, such that transcription and protein degradation could be modeled in the same equation. Additionally, it was assumed that GTL1 and RSL4 proteins degrade linearly.

The gene regulatory network and experimental data predicted that GTL1 represses RSL4. Thus, a Hill equation was used to model RSL4 transcription, where increased levels of GTL1 result in decreased levels of RSL4. As the oligomeric state of GTL1 is unknown, there was a possibility of GTL1 forming higher oligomers, where *n*_1_ is the number of GTL1 proteins bound to each other.

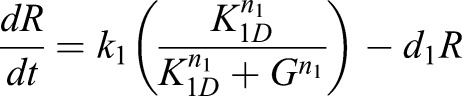
The gene regulatory network predicted that RSL4 activates GTL1. In addition, the experimental data suggested that GTL1 represses itself. Thus, the Hill equation for GTL1 takes into account what happens when both RSL4 and GTL1 bind the TL1 promoter at the same time. One possibility is that GTL1 inhibition overcomes the RSL4 activation. This means that, in order for GTL1 to be transcribed, GTL1 must be unbound (Eqn 1). In the other case, RSL4 activation could overcome GTL1 inhibition. This means that, as long as RSL4 is bound, GTL1 will be transcribed, even if GTL1 is bound to its own promoter (Eqn 2). As in the RSL4 equation, there is a possibility of higher oligomeric states.
(1)
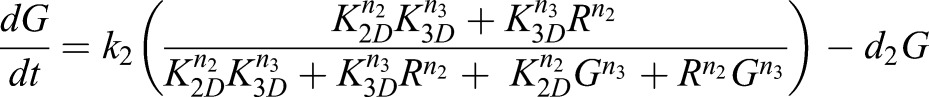

(2)
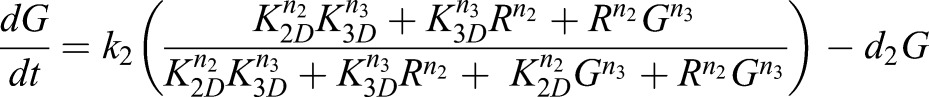


After constructing the two models, nullcline analysis was performed to check for steady-state solutions. Mathematica was used to analytically solve for the nullclines as well as for steady states. As concentrations must be positive values, only the region where *R*≥0 and *G*≥0 was considered. In this region, the model with Eqn 1 has no steady states, whereas the model with Eqn 2 has one steady state. In a wild-type case, there should be a steady state value of *R* and *G* that produces root hairs with normal length. Thus, the model with Eqn 2 was used for the simulations.

A sensitivity analysis was used to identify the parameters that most greatly affect the model outcome, as these parameters could give insight into the effects of RSL4 and GTL1 mutants and overexpression lines. Sobol decomposition, which quantifies sensitivity by calculating the variance in the model outcome as the parameters are changed (Sobol, 2001), was used to calculate the sensitivity indices. The index was calculated for each parameter using 1000 Monte Carlo evaluations and repeated 10 times for technical replicates (Clark et al., 2016).

## Supplementary Material

Supplementary information

Supplementary information
