# Supplementary material for: GTL1 and DF1 regulate root hair growth through transcriptional repression of ROOT HAIR DEFECTIVE 6-LIKE 4 in Arabidopsis
Source: Development. 2018 Feb 1;145(3):dev159707. doi: 10.1242/dev.159707 (PMC5818008; doi:10.1242/dev.159707)
Supplement: Supplementary information [file develop-145-159707-s1.pdf]

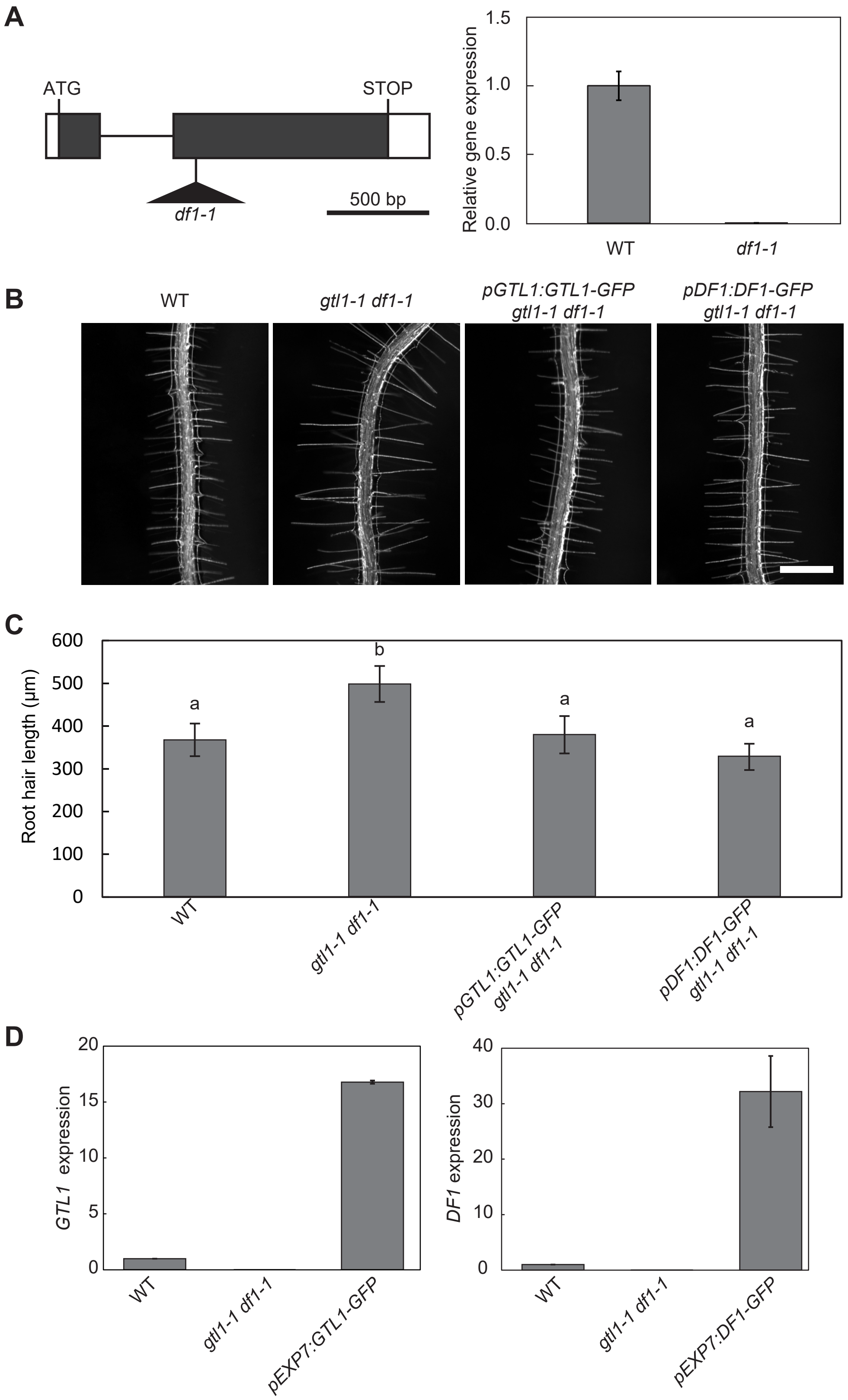
A
ATG
STOP
df1-1
500 bp
Relative gene expression
1.5
1.0
0.5
0.0
WT
df1-1
B
WT
gtl1-1 df1-1
pGTL1:GTL1-GFP
gtl1-1 df1-1
pDF1:DF1-GFP
gtl1-1 df1-1
C
Root hair length (μm)
600
500
400
300
200
100
0
a
b
a
a
WT
gtl1-1 df1-1
pGTL1:GTL1-GFP
gtl1-1 df1-1
pDF1:DF1-GFP
gtl1-1 df1-1
D
GTL1 expression
20
15
10
5
0
WT
gtl1-1 df1-1
pEXP7:GTL1-GFP
DF1 expression
40
30
20
10
0
WT
gtl1-1 df1-1
pEXP7:DF1-GFP

Fig. S1. Molecular genetic characterization of *df1-1*, *gtl1-1 df1-1*, *pEXP7:GTL1-GFP* and *pEXP7:DF1-GFP* plants

(A) Structure of DF1 gene. Black boxes represent exons and a black arrowhead marks the position of T-DNA insertion in *df1-1* (SALK_106258). RT-qPCR analysis of WT and *df1-1*. Expression levels are normalized against those of *UBQ10*. Data are mean ± SD (n = 3, biological replicates). **(B)** Root hair phenotypes of WT, *gtl1-1 df1-1*, *gtl1-1 df1-1 pGTL1:GTL1-GFP* and *gtl1-1 df1-1 pDF1:DF1-GFP* seedlings. Bar = 500 µm. **(C)** Quantitative analysis of root hair length. Data are mean ± SE (n = 120). Different letters indicate means that differ significantly (Turkey-Kramer test, $P < 0.01$). Note that introduction of *pGTL1:GTL1-GFP* or *pDF1:DF1-GFP* complements the root hair phenotype in *gtl1-1 df1-1*. (**D**) RT-qPCR analysis of WT, *gtl1-1 df1-1*, *pEXP7:GTL1-GFP* and *pEXP7:DF1-GFP*. Expression levels are normalized against those of *UBQ10*. Data are mean ± SD (n = 3, biological replicates).

Development • Supplementary information

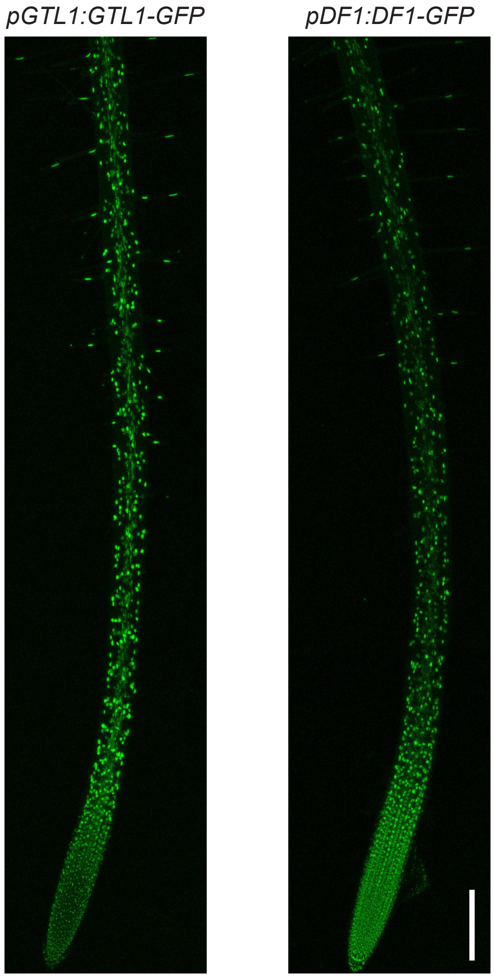

Fig. S2. Confocal images of GTL1-GFP and DF1-GFP in *pGTL1:GTL1-GFP* and *pDF1:DF1-GFP* roots.

Images are produced by maximum intensity projection from 21 Z-stack images and merged from multiple images. Bar = 250 μm.

Development • Supplementary information

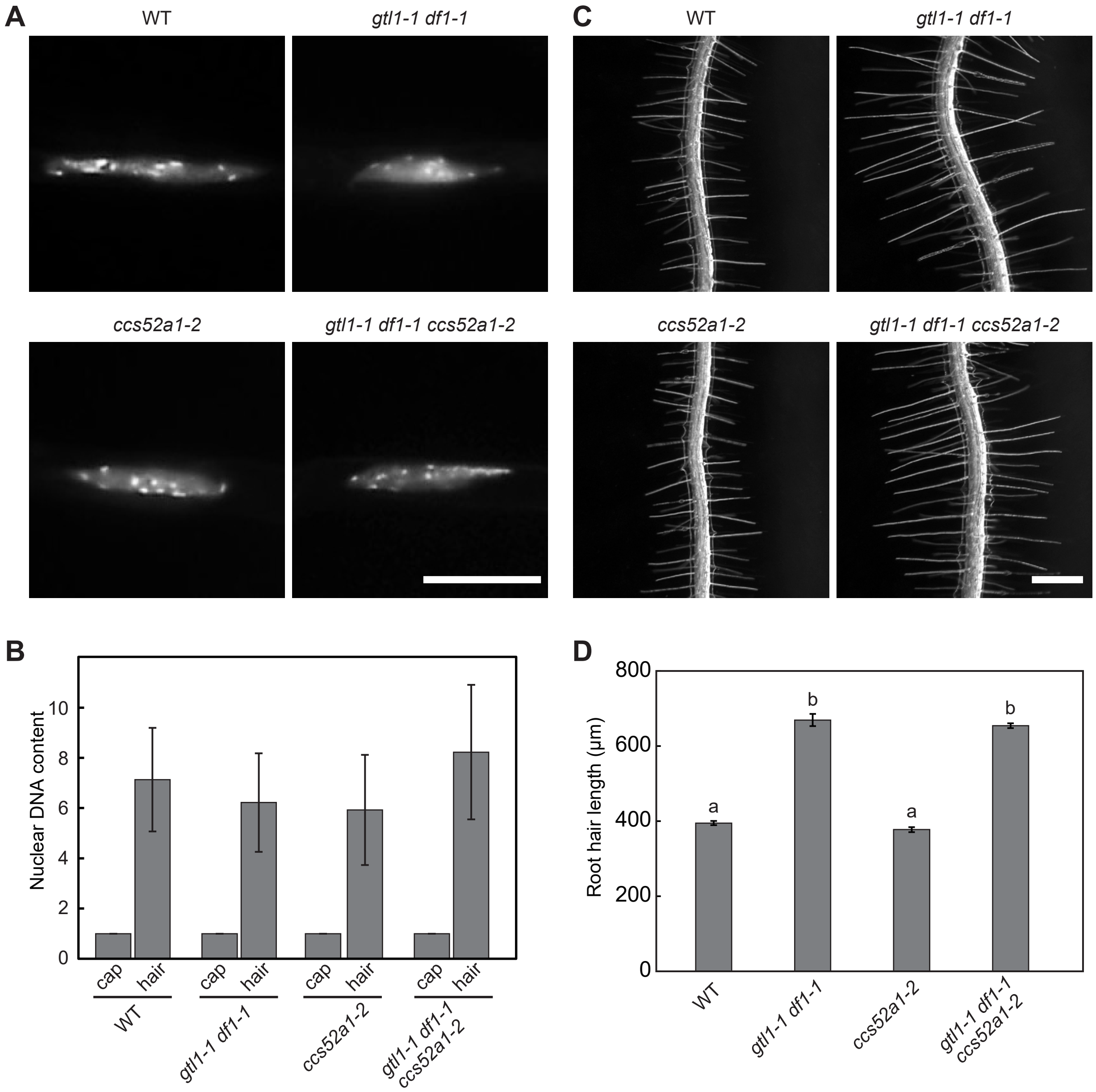

Fig. S3. GTL1 and DF1 regulate ploidy-independent root hair growth

(A) DAPI-stained nuclei in mature root hairs of WT, *gtl1-1 df1-1*, *ccs52a1-2* and *gtl1-1 df1-1 ccs52a1-2* seedlings. Bar = 20 µm. **(B)** Quantitative analysis of nuclear DNA content in WT, *gtl1-1 df1-1*, *ccs52a1-2* and *gtl1-1 df1-1 ccs52a1-2* root hairs. Nuclear DNA content in root cap cells was used as a reference. Data are mean ± SE ($n > 40$ for root caps and $n > 120$ for root hairs). **(C)** Root hair phenotypes of WT, *gtl1-1 df1-1*, *ccs52a1* and *gtl1-1 df1-1 ccs52a1* seedlings. Bar = 500 µm. **(D)** Quantitative analysis of root hair length. Data are mean ± SE ($n = 120$). Different letters indicate means that differ significantly (Turkey-Kramer test, $P < 0.05$).

Development • Supplementary information

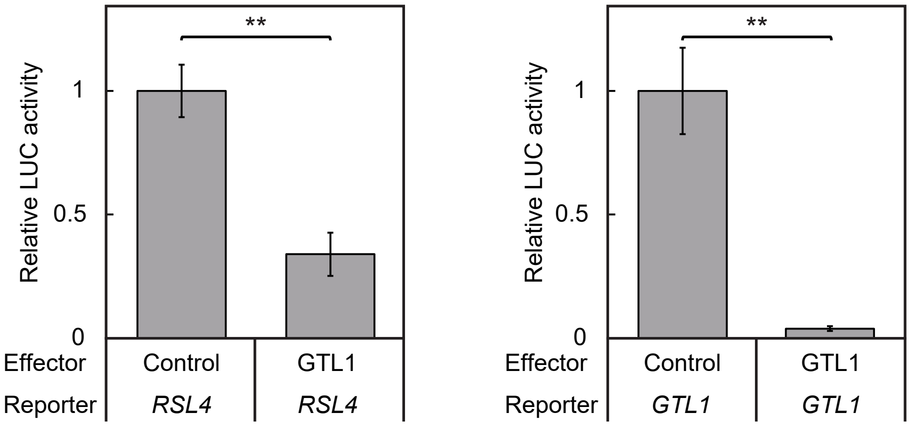

Fig. S4. GTL1 represses the *RSL4* and its own promoter in Arabidopsis cell culture

The promoter-luciferase assay in Arabidopsis MM2D culture cells. Data are mean ± SD (n = 3). Asterisks indicate a significant difference compared to vector control (Student' s t-test, ** $P < 0.01$).

Development • Supplementary information

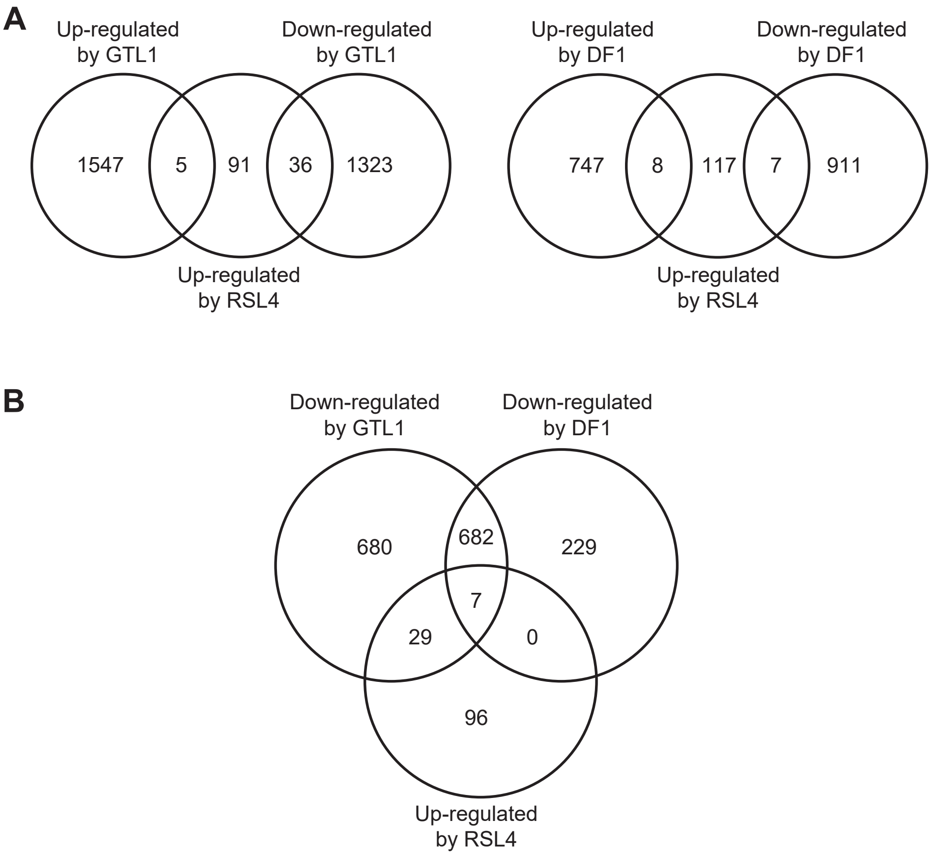

C

Description	p-value
translation	8.30E-15
cellular macromolecule biosynthetic process	1.03E-14
macromolecule biosynthetic process	1.03E-14
cellular protein metabolic process	3.04E-14
biosynthetic process	1.09E-11
cellular biosynthetic process	8.20E-11
protein metabolic process	8.20E-11
cellular macromolecule metabolic process	2.57E-10
gene expression	1.96E-09
cellular metabolic process	3.94E-09
metabolic process	3.85E-08
primary metabolic process	4.80E-08
macromolecule metabolic process	7.75E-08
cellular process	3.36E-07
response to stimulus	1.65E-03
response to temperature stimulus	3.31E-03
response to cold	3.93E-03
response to abiotic stimulus	1.75E-02
response to chemical stimulus	3.14E-02
signaling pathway	4.83E-02
response to stress	4.83E-02
carbohydrate biosynthetic process	4.83E-02

Development • Supplementary information

Fig. S5. Genes commonly regulated by GTL1, DF1 and RSL4 in root hairs

(A) Venn diagrams between 132 genes up-regulated by RSL4 and genes up- or down-regulated by GTL1 (left) and DF1 (right) in root hairs. GTL1-GFP and DF1-GFP expressing root hairs were isolated by cell sorting from *pEXP7:GTL1-GFP* and *pEXP7:DF1-GFP* seedlings, respectively. Root hairs in *gtl1-1 df1-1* were isolated by sorting GFP-expressing cells in *gtl1-1 df1-1* seedlings transformed with *pEXP7:NLS-GFP*. Genes up- or down-regulated by GTL1 include those differentially expressed more than 2-fold between *pEXP7:GTL1-GFP* and *gtl1-1 df1-1* root hairs with cut-off *P*-values of 0.05. Genes up- or down-regulated by DF1 include those differentially expressed more than 2-fold between *pEXP7:DF1-GFP* and *gtl1-1 df1-1* root hairs with cut-off *P*-values of 0.05. RSL4 induced genes include 84 genes identified in Yi et al. (2010), 29 genes identified in Vijayakumar et al. (2016) and 11 genes identified in Won et al. (2009). **(B)** Venn diagram analysis highlights 36 genes commonly regulated by GTL1 and RSL4, 7 of which are also regulated by DF1. **(C)** Gene Ontology terms over-represented among 689 genes commonly regulated by GTL1 and DF1.

Development • Supplementary information

A

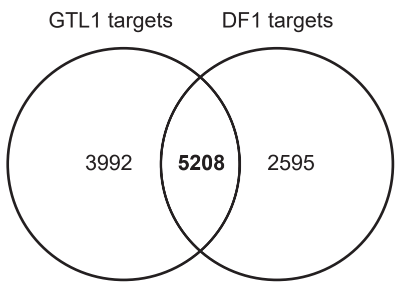

B

Description	p-value
response to chemical stimulus	4.39E-33
response to organic substance	1.55E-29
response to stimulus	1.41E-27
response to endogenous stimulus	3.09E-20
regulation of cellular metabolic process	1.70E-18
regulation of biosynthetic process	1.83E-18
regulation of cellular biosynthetic process	1.83E-18
regulation of transcription	3.79E-18
regulation of nitrogen compound metabolic process	4.89E-18
response to hormone stimulus	5.74E-18
regulation of macromolecule biosynthetic process	5.74E-18
regulation of primary metabolic process	8.28E-18
regulation of nucleic acid metabolic process	9.98E-18
regulation of metabolic process	4.10E-16
regulation of gene expression	3.30E-15
regulation of macromolecule metabolic process	1.15E-14
response to stress	3.06E-14
regulation of cellular process	1.01E-13
response to chitin	1.46E-13
response to abiotic stimulus	1.60E-13

Fig. S6. Genes directly bound by GTL1 and DF1 in Arabidopsis roots

(A) Venn-diagram analysis of putative GTL1 and DF1 direct targets. Promoter binding of GTL1 and DF1 is based on ChIP-chip analysis using *pGTL1:GTL1-GFP* and *pDF1-DF1-GFP* roots with a cut-off Z-score of 1.5. **(B)** Gene Ontology terms over-represented among 5208 genes directly bound by GTL1 and DF1.

Development • Supplementary information

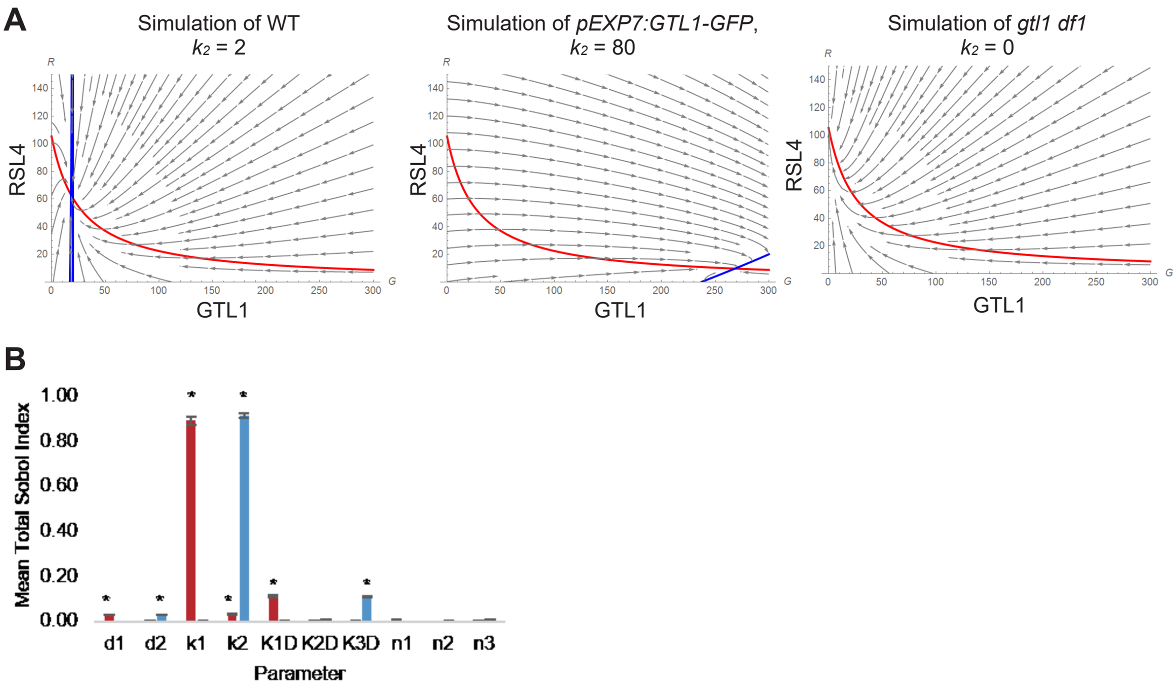

Fig. S7. Sensitivity analysis and phase-plane analysis of mathematical model

(A) Phase plane analysis of the mathematical model in WT (left), *pEXP7:GTL1-GFP* (center), and *gtl1-1 df1-1* mutant (right) cases. Red and blue lines represent the nullclines for the RSL4 and GTL1 equations, respectively. Grey arrows represent the vector field. In each phase plane, the nullclines intersect at one equilibrium point. The arrows point towards the equilibrium, showing that this is a stable steady state solution to the mathematical model. **(B)** Sensitivity analysis of parameters in the mathematical model. In each simulation, parameters were varied across 1000 randomly selected values. The total Sobol index was calculated for each parameter in each simulation. Red and blue bars represent the indices for the RSL4 and GTL1 equations, respectively. Error bars represent SEM (n = 10 simulations). Asterisks denote parameters that have a significantly higher total Sobol index (Wilcoxon with Steel-Dwass, $\alpha = 0.05$).

Development • Supplementary information

Table S1. A list of genes transcriptionally regulated by GTL1 and DF1 in Arabidopsis root hairs

Click here to Download Table S1

Table S2. A list of genes directly bound by GTL1 and DF1 in Arabidopsis roots

Click here to Download Table S2

Table S3. Effects of edge threshold on precision of inferred GRN

Precision of network given a different maximum number of edges. The threshold was used to select the maximum number of edges. The threshold with the highest precision was used in the gene regulatory network pipeline.

Threshold	Total number of edges	Number of edges from GTL1	Number of edges from DF1	True positives (TP)	False positives (FP)	Precision (TP/(TP+FP)
floor(1.85*number of genes)	66	32	5	12	25	0.324324324
floor(1.8*number of genes)	64	32	3	12	23	0.342857143
floor(1.75*number of genes)	63	32	2	12	22	0.352941176
floor(1.7*number of genes)	61	32	1	12	21	0.363636364
floor(1.65*number of genes)	59	30	1	12	19	0.387096774
floor(1.6*number of genes)	57	28	1	11	18	0.379310345
floor(1.55*number of genes)	55	28	0	10	18	0.357142857

Table S4. Parameter values used in the model

Parameter values and initial conditions used in model simulations. The parameters given are for the wild type condition. For GTL1 overexpression and mutants, the only parameter value changed was k_2.

Parameter	Biological meaning	Value
d_1	Degradation rate of RSL4	0.095 time units^{-1}
d_2	Degradation rate of GTL1	0.1 time units^{-1}
k_1	Production rate of RSL4	10 time units^{-1}
k_2	Production rate of GTL1	2 time units^{-1}
K_{1D}	Dissociation constant for GTL1 binding RSL4 promoter	27 concentration units
K_{2D}	Dissociation constant for RSL4 binding GTL1 promoter	100 concentration units
K_{3D}	Dissociation constant for GTL1 binding its own promoter	100 concentration units
n_1	Oligomeric state of GTL1 when it binds RSL4 promoter	1 (unitless)
n_2	Oligomeric state of RSL4 when it binds GTL1 promoter	1 (unitless)
n_3	Oligomeric state of GTL1 when it binds its own promoter	1 (unitless)

Development • Supplementary information

Table S5. A list of primers used in this study

name	seq	note	referece
WF627-F1	TTCTCGTCTCATAGCTCATCG	For genotyping of *gtl1-1*	Breuer et al 2009
WF627-R1	TGGCCATCTTGATGATGATGG		Breuer et al 2009
DF1_258-F1	TGCTGATTGATCCACTTCTCA	For genotyping of *df1-1*	
DF1_258 R5	GAGATGTTTGGAAACGAAGGTAC		
rsl4-1 homozygote F	GAAAGCTTCGGTCACAAGTGTTAAA	For genotyping of *rsl4-1*	Yi et al 2010
rsl4-1 LB R	TTGTAAGCCAATGGTGCGTACAT		Yi et al 2010
JIC-RB1	CCGAACAAAAATACCGGTTCCC		
Wis1	AACGTCCGCAATGTGTTATTAAGTTGTC	For T-DNA of Wisconsin lines	
LBa1	TGGTTCACGTAGTGGGCCATCG	For T-DNA of SALK lines	
wind farm promoter F	CACCCACCTTCTTCTTCTTCTTCACCTTC	For cloning of *GTL1* from promoter to stop	
AT1G33240lost TAA	CTGAACCATTGTCAAGAAAGGTGG		
DF1p FW cacc	CACCGACTTCCATCTAACCACTTTCCTTCC	For cloning of *DF1* from promoter to stop	
DF1 stop R without TAA	CAGATTATTCGTCGTCTTGTTG		
GTL1-627F2	ATGGAATTGTTTGAAGGTTTGG	For qPCR of *GTL1*	Breuer et al 2009
GTL1-627R2	GACATGACCTCGTGTTCTCG		Breuer et al 2009
DF1F5KN	GACATGGGAATAGCGTTTCG	For qPCR of *DF1*	
DF1R4KN	TCGGCATAACTGTCGTTACC		
RSL1_FWD	TCGTACCGCTACTCGGCTTCTT	For qPCR of *RSL1*	Rymen et al 2017
RSL1_REV	CAATAAACGGCCTTTCACGGGAGA		Rymen et al 2017
RSL2_FWD	CTCGTCCCCAATGGAACAAAGGTC	For qPCR of *RSL2*	Rymen et al 2017
RSL2_REV	GCAATCGGCGCATACATCCATAGA		Rymen et al 2017
RSL3_FWD	TCGTCCCTAATGGAACAAAGGTTG	For qPCR of *RSL3*	Rymen et al 2017
RSL3_REV	GGCCAATGTCCATTCCGTTGTAAG		Rymen et al 2017
qRSL4-F	AGGCAAAACTAGAGCCACCA	For qPCR of *RSL4*	
qRSL4-R	ATCGACTTTTGTCCCGTTTG		
qRHD6-F	TCACGAGAGCTTTCCTCCTC	For qPCR of *RHD6*	
qRHD6-R	TGAAGCCGTAGCTCATGTTG		
qIPS2-F	GAGCGATGAAGATTGCATGA	For qPCR of *IPS2*	
qIPS2-R	CCGGAAACAAAGTAAACACG		
UBQ10-F	GAAGTGGAAAGCTCCGACAC	For qPCR of *UBQ10*	Shibata et al 2013
UBQ10-R	TTAGAAACCACCACGAAGACG		Shibata et al 2013
RSL4-0-F	TGGAGTCTCTCTTGGGGATG	For ChIP-qPCR of *RSL4* promoter	
RSL-0-R	GGAGCAAGAAAGCATGATGA		
RSL4-500-F	TTCACCGCTTCTCTTGTGTG	For ChIP-qPCR of *RSL4* promoter	
RSL4-500-R	ACAGGCTTTGTTCCTTCACG		
RSL4-1000-F	TGTTGAATTCGCCTTATTTGC	For ChIP-qPCR of *RSL4* promoter	
RSL4-1000-R	TTCGTTTGTTCATGTGTTAAAGTG		
RSL4-1500-F	ATGTACCGTAACCCGTCCTC	For ChIP-qPCR of *RSL4* promoter	
RSL4-1500-R	GACGGCACAGAACAGAGTTG		
TA3-FW	CTGCGTGGAAGTCTGTCAAA	For ChIP-qPCR of *TA3*	Yamaguchi et al 2014
TA3-RV	CTATGCCACAGGGCAGTTTT		Yamaguchi et al 2014
NcoI WFp1.8 FW	AGCTAACCATGGCACCTTCTTCTTCTTCTTCACCTTC	For Luc assay of *GTL1* promoter	
5UTR RV+NotI	AGCTAAGCGGCCGCCAATCTTGATGATGTTTCAATC		
SacII RSL4pro Fw	AAAGTAGCTAACCCGCGGGTGTGTGCATGCATGTGTGT	For Luc assay of *RSL4* promoter	Rymen et al 2017
BamHI RSL4pro RV	AAATTAAAGCTAACGGATCCCGCTCTAACTGATCAACTCTTGCC		Rymen et al 2017
SmaI-GTL1start-F	TAATTCCCGGGATGGAGCAAGGAGGAGGTG	For Luc assay of *GTL1* coding region	
SmaI-GTL1stop-R	ATTTACCCGGGTTACTGAACCATTGTCAAGAAAG		

Development • Supplementary information
